# Investigation of *ELF5*,* KIF18A*,* NPTX1* and *COL23A1* genes in the pathophysiology of indirect inguinal hernia in children

**DOI:** 10.1007/s00383-026-06400-y

**Published:** 2026-04-02

**Authors:** Ercan Genc, Tugay Tartar, Ebru Onalan, Unal Bakal, Mehmet Sarac, Tugce Kaymaz, Ahmet Kazez

**Affiliations:** 1 Afyonkarahisar State Hospital, Afyonkarahisar, Turkey; 2 Department of Pediatric Surgery, Firat University Faculty of Medicine, 23119 Elâzığ, Turkey; 3 Adıyaman University, Adıyaman, Turkey; 4 Nevşehir Hacı Bektaş Veli University, Nevşehir, Turkey

**Keywords:** Inguinal hernia, Etiology, Genetics, Polymorphism, Pediatric

## Abstract

**Purpose:**

In this study, we investigated the effect of *ELF5*,* KIF18A*,* NPTX1* and *COL23A1* genes in residual processus vaginalis (PV) the main factor in the development of indirect inguinal hernia (IIH) in children.

**Methods:**

Cases operated for IIH in children aged 0–18 years between 2018 and 2021 constituted the study group, and cases with undescended testis without hernia the control group. Protein levels of *KIF18A*, which has the highest gene expression in tissue samples, were also analyzed by ELISA method. Polymorphisms of *ELF5*,* KIF18A* and *COL23A1* genes with the highest level of expression change in blood and tissue samples were analyzed by qRT-PCR. Statistical analysis was performed.

**Results:**

There were 186 patients in the study group and 26 patients in the control group. The results of *ELF5*,* KIF18A* and *COL23A1* genes were significantly higher in the study group than in the control group. There was a statistically significant positive correlation between *GAPDH* and *ELF5*, *KIF18A* and *NPTX1* in the study group. Also, a statistically significant positive correlation was found between *KIF18A* and *ELF5* and between *NPTX1* and *ELF5* and *COL23A1*.

**Conclusion:**

The present study may be the first study conducted in human tissue samples that we could access in the literature in terms of the genetic factors. It was predicted that *ELF5*,* KIF18A* and *COL23A1* genes may be counted among the effective factors in PV closure. This study may shed light on larger prospective genetic studies.

## Introduction

Almost all inguinal hernias (IH) in children are indirect inguinal hernias (IIH). Factors predisposing to IH in children include: prematurity, low birth weight, family history, hydrops fetalis, meconium peritonitis, chylous ascites, urinary ascites, ascitic liver diseases, intersex anomalies, exstrophy vesica, omphalocele and gastroschisis, undescended testis, cystic fibrosis, mucopolysaccharidosis, connective tissue diseases, hip dislocation, ventriculoperitoneal shunts and continuous peritoneal dialysis [1].

The patency of processus vaginalis (PV) is the main mechanism in the development of IIH. Studies on genetic factors among the causes of unclosed PV are very few in the literature. In one study, it was shown that the risk of IH increased in children of parents with a history of IH [2]. There are studies suggesting that *GATA6*, which is one of the transcription factors that play an important role in embryologic development, and *TBX3* gene variants that play a role together with G*ATA6* in the protection of the *self-renewing* feature of embryonic stem cells and in the differentiation process are effective in the development of IH [3, 4]. Increased *Insl3* gene expression which has been shown to be involved in gubernacular development (due to causing collagen structure disorder), *SIRT1* gene misregulation which is involved in fibroblast proliferation and a polymorphism found in *COL1A1* gene which is involved in type 1 collagen formation have also been shown to be involved in the development of IH [5, 6]. Du et al. found that genetic variants in *ELF5*, *KIF18A*, *COL23A1* in *Sus scrofa chromosomes (SSC) 2 and NPTX1* region in *SSC 12* were effective in the development of IH in pigs [7].

The aim of this study was to investigate the effect of polymorphic variants of *ELF5*, *KIF18A*, *NPTX1* and *COL23A1* genes in PV residuals on the etiology of IIH in children.

## Materials and methods

The study included 186 children aged 0–18 years with IH (Study group) and 26 children without a history of IH who underwent surgery for undescended testis (Control group) between January 2018 and January 2021 at the Pediatric Surgery Clinic of Firat University Hospital. Peripheral blood and tissue samples were obtained from all patients. Tissue samples were obtained from PV tissue in the study group. In the control group, tissue samples were obtained from the obliterated parietal peritoneal remnants following the release of the testes and their appendages into the peritoneal cavity. Molecular and biochemical analyses were performed in the Laboratory of the Department of Medical Biology, Faculty of Medicine, Firat University.

### Molecular genetic studies

####  RNA isolation

Tissue samples obtained from the patients were stored in ependorf tubes at −80 °C until use. 500 µL of trizol solution was added to 0.3–0.4 g of weighed tissue. The tissues were disintegrated with the help of beads in a Next Advance homogenization device. Tissues were homogenized in the homogenizer at 8000 rpm for 5 min. After waiting at room temperature for 5 min, the supernatant was taken from the top after centrifugation at 12.000 rpm for 2 min, transferred to new tubes, 100 µL chlorophane was added and vortexed. After waiting at room temperature for 2–3 min, the supernatant was centrifuged at 12.000 rpm for 15 min at 2–4 °C. The liquid in the tube was separated into 3 phases, the uppermost liquid phase was transferred to another tube and 250 µL isopropyl alcohol was added and vortexed. After waiting at room temperature for 10 min, centrifuged at 12.000 rpm for 10 min at 2 °C. The supernatant was poured off, the remaining pellet was washed by adding 500 µL 75% ethanol, washed upside down, and centrifuged at 7,500 rpm for 5 min at 2 °C. The process was repeated. After drying, 30 µL of water was added, pipetted and stored at −40 °C. RNA concentrations were measured with a nanodrop device (Allsheng Nano-400 A Hangzhou. China).

#### cDNA synthesis

For the cDNA synthesis reaction, 1 µl reaction buffer, 0.5 µl dNTP (doexyribonucleotide triphosphate; deoxyribonucleotide triphosphates) mixture, 1 µl random hexamer, 0.25 µl reverse transciptase (RT) enzyme inhibitor, 0.5 µl RT enzyme and 1.75 µl RNase-free water were added to the tubes to make a 10 µl volume of reaction solution.

#### Quantitative Real Time Polymerase Chain Reaction (qRT-PCR)

The mRNA expression of *ELF5*, *KIF18A*, *NPTX1*, *COL23A1* genes was determined in tissue samples by Real-Time Quantitative Reverse Transcription polymerase chain reaction (qRT-PCR). primer sequences to evaluate the mRNA levels of the genes were determined using the web address www.origene.com. A one-sample mix for qRT-PCR was prepared using 0.5 µl cDNA, 2.5 µl master mix, 0.05 µl each of 10 pmol R and F primers, 0.3 µl ROX dye and 1.6 µl nuclease-free water in a total volume of 5 µl. The RT-PCR reaction was performed using Applied Biosystems 7500 Software v2.0.5, Waltham, Massachusetts, USA for a total of 40 cycles of 10 min at 95 °C in the holding cycle and seconds at 95 °C and 1 min at 60 °C in the cycling cycle. *GAPDH* was used as a control gene. Each sample was run in 3 replicates. The 2-∆∆∆CT method was used to calculate the differences in gene expression as a result of qRT-PCR analysis. Table 1 lists the primers used in qRT-PCR.

**Table 1:** Primer sequences used in qRT-PCR (www.origene.com)

Gene name	Nucleotide sequence (5’−3’)
*ELF5* (Forward)	TGGACTCCGTAACCCATAGCACCT
*ELF5* (Reverse)	ATTGCTTAAGGGCTGATGGCATCG
*KIF18A* (Forward)	CAGTTCAGCCTATTCCTT
*KIF18A* (Reverse)	TATCACTGTTTATGTTTGAGC
*NPTX1* (Forward)	CTACACCAGCGGATCAGCGA
*NPTX1* (Reverse)	CTCCGCCTAGAGTGTCCTGC
*COL23A1* (Forward)	AGGTTCTTACAGGGCAGTA
*COL23A1* (Reverse)	TAACATTCAAGAGTATGCCCACC
*GAPDH* (Forward)	GTCTCCTCTGACTTCAACAGCG
*GAPDH* (Revers)	ACCACCCTGTTGCTGTAGCCAA

#### Enzyme-Linked Immunosorbent Assay (ELISA) analysis

50 mg of hernia tissue was homogenized (Bullet Blender Storm 24, New York, USA) by adding 450 µl PBS (phosphate buffered saline) and 10 µl Protease Inhibitor Cocktail (Abcam, Cat No: ab65621). After homogenization, the samples were centrifuged at 4,000 rpm for 10 min at 4 °C (Beckman Coulter Allegra X-30R Centrfifuge, Indianapolis, USA) and the supernatants were used for ELISA analysis. *KIF18A* protein concentration was tested by ELISA kit (human *KIF18A*; catalog no: DZE201122778; SunRed, Shanghai, China). The determination of *KIF18A* protein concentration followed the manufacturer’s sandwich ELISA protocol. The measurement range of the human *KIF18A* kit is 3–48 ng/ml.

### Statistical analysis

The data of the patients included in the study were evaluated with the University’s licensed analysis program SPSS (Statistical Package for Social Sciences; SPSS Inc., Chicago, IL) 22 package program. Descriptive data were presented as n, % values for categorical data and mean±standard deviation (Mean ± SD) and median interquartile range (25–75 percentile values) for continuous data. Chi-square analysis (Pearson Chi-square) was used to compare categorical variables between groups. The conformity of continuous variables to normal distribution was evaluated by Shapiro Wilk test. Mann Whitney U-test was used for comparison of paired groups. Spearman correlation analysis was performed to determine the relationship between the measured data. Statistical significance level was accepted as *p* < 0.05.

## Results

### Demographic and clinical characteristics

A total of 212 patients were included in the study, comprising 186 patients diagnosed with IH (study group) and 26 patients with undescended testis (control group). The demographic and clinical characteristics of the participants are summarized in Table 2.

Regarding gender distribution, the study group was predominantly male (*n* = 166, 89.2%), while all patients in the control group were male (*n* = 26, 100%). The difference in gender distribution between the groups was not statistically significant (*p* = 0.143). A positive family history of IH in first-degree relatives was reported in 8.6% (*n* = 16) of the study group and 15.4% (*n* = 4) of the control group, with no statistically significant difference observed (*p* = 0.280).The mean age at surgery was significantly lower in the study group (2.32 ± 3.54 years) compared to the control group (2.90 ± 4.10 years) (*p* = 0.029), suggesting that patients with IH underwent surgical intervention at a younger age.

Regarding laterality, right-sided involvement was most common in both groups (48.4% in the study group vs. 50% in the control group). Left-sided cases were observed in 37.1% of the study group and 26.9% of the control group, while bilateral involvement was found in 14.5% and 23.1%, respectively. No significant difference in side distribution was noted (*p* = 0.418). Prematurity was present in 19.4% of the study group and 11.5% of the control group, with no significant difference (*p* = 0.427). Cardiac disease was observed in 10.8% of the study group and 3.8% of the control group, while neurological disorders were found in 10.2% and 11.5%, respectively. Umbilical hernia (8.1%) and undescended testicle (4.3%) were seen only in the study group. No statistically significant difference was found between groups regarding comorbidities (*p* = 0.193).

In summary, the two groups were largely similar in terms of gender distribution, family history, prematurity, laterality, and comorbidities. The only statistically significant difference was the mean age at surgery, which was lower in the IH group.

**Table 2:** Demographic characteristics of the groups

		Study group *n*, %	Control group *n*, %	*p* value
Gender	Male	166 (89.2)	26 (100)	0,143
	Female	20 (10.8)	-	
Side	Right	90 (48.4)	13 (50)	0.418
	Left	69 (37.1)	7 (26.9)	
	Bilateral	27 (14.5)	6 (23.1)	
Additional disease	Cardiac disease	20 (10.8)	1 (3.8)	0.193
	Neurological disorder	19 (10.2)	3 (11.5)	
	Umbilical hernia	15 (8.1)	-	
	Undescended testis	8 (4.3)	-	
Presence of IH in 1 st degree relative		16 (8.6)	4 (15.4)	0.280
Average age at surgery		2.32 ± 3.54	2.90 ± 4.1	0.029*
Prematurity		36 (19.4)	3 (11.5)	0.427

Statistical significance are bold fonts

*IH* Inguinal hernia

*Statistically significant level *p <* 0.05

## Gene expression analysis

The comparative analysis of gene expression profiles between the study and control groups revealed significant differences in several target genes (Table 3). Notably, A statistically significant upregulation was observed in the study group for *ELF5*, *KIF18A*, and *COL23A1* gene expressions. In contrast, no statistically significant differences were found for *NPTX1* expression between groups (median: 1.09 vs. 1.10; *p* = 0.712). Similarly, *GAPDH*, used as a housekeeping gene, showed no significant variation (*p* = 0.310), confirming the stability of internal normalization and supporting the reliability of the gene expression analysis.

### Protein level analysis

Despite the significant transcriptional upregulation of *KIF18A*, tissue *KIF18A* protein levels, as measured by ELISA, did notshow a significant difference between the study and control groups (median: 12.40 vs. 12.28; *p* = 0.972). This discrepancy between mRNA and protein levels may indicate the involvement of post-transcriptional regulatory mechanisms, differential protein turnover, or limited systemic reflection of tissue-specific gene expression changes.

### Overall pattern

Collectively, these findings demonstrate a selective upregulation of specific genes (*ELF5*,* KIF18A*,* COL23A1*) in the study group, while *NPTX1* remained unchanged. The lack of difference in *KIF18A* protein levels further underscores the complexity of gene–protein regulatory dynamics in the studied condition. A detailed comparison of the genetic characteristics between the groups is presented in Table 3.

**Table 3:** Comparison of genetic characteristics of the groups

Genes	Detection method	Study group median (min-max)	Control group median (min-max)	*p*
*ELF5*	qRT-PCR	5.79 (0.00–10953590029.00.00.00)	0.90 (0.00–114.70.00.70)	< 0.001*
*KIF18A*	qRT-PCR	7.94 (0.00–164555880.50.00.50)	0.75 (0.00–79.80.00.80)	0.004*
*NPTX1*	qRT-PCR	1.09 (0.00–10009316094.00.00.00)	1.10 (0.00–57.30.00.30)	0.712
*COL23A1*	qRT-PCR	3.73 (0.02–199.52.02.52)	1.05 (0.00–29.70.00.70)	< 0.001*
*GAPDH*	qRT-PCR	24.50 (17.40–28.00)	25.50 (20.40–27.80)	0.310
*KIF18A*	ELISA	12.40 (6.15–31.93)	12.28 (2.71–30.88)	0.972

Statistical significance are bold fonts

*Statistically significant level *p <* 0.05

### Correlation analysis of gene expression in the study group

Correlation analyses were performed to assess gene expressionrelationship within the study group, with results summarized in Table 4. A significant positive correlation was found between *KIF18A* and *ELF5* (*r* = 0.211, *p* = 0.044), suggesting potential co-regulation or shared pathways. No significant correlation was observed between *KIF18A* and *NPTX1* (*r* = 0.157, *p* = 0.093), or *KIF18A* and *COL23A1* (*r* = 0.004, *p* = 0.964). However, *NPTX1* showed significant positive correlation with both ELF5 (*r* = 0.247, *p* = 0.003) and *COL23A1* (*r* = 0.303, *p* < 0.001), indicating potential interaction among these genes in the pathophysiology of IH. No significant correlation was identified between *ELF5* and *COL23A1* (*r* = 0.088, *p* = 0.366). Overall, the correlation matrix reveals partial clustering of *ELF5*, *KIF18A*, and *NPTX1* expression, while *COL23A1* appears more selectively associated with *NPTX1* and not with *GAPDH* or *KIF18A*.

### Gender-based comparison of gene expression

In the study group, gene expression levels were analyzed by gender. No statistically significant differences were found between male and female patients for *ELF5* (*p* = 0.902), KIF18A (*p* = 0.107), *NPTX1* (*p* = 0.183), *COL23A1* (*p* = 0.820), or *GAPDH* (*p* = 0.222). Similarly, tissue *KIF18A* protein levels, measured by ELISA, did not differ significantly between genders (*p* = 0.776). These findings suggest that the observed gene expression alterations in the study group are unlikely to be influenced by sex-based biological differences.

**Table 4:** Correlation of genetic characteristics of the patient group

Genetic correlation		*GAPDH*	*KIF18A*	*NPTX1*	*COL23A1*
*KIF18A*	*r*	**0.260**			
	*p*	0.003*			
*NPTX1*	*r*	**0.391**	0.157		
	*p*	0.000*	0.093		
*COL23A1*	*r*	− 0.004	0.004	**0.303**	
	*p*	0.962	0.964	0.000*	
*ELF5*	*r*	**0.417**	**0.211**	**0.247**	0.088
	*p*	0.000*	0.044*	0.003*	0.366

Statistical significance are bold fonts

*Statistically significant level *p <* 0.05

## Discussion

Almost all IHs in children are indirect hernias. Since the occurrence of IH is directly related with the descent of the testicle from the abdomen to the scrotum, it is 4–20 times more common in boys compared to girls [8]. IHs are observed in 1–3% of children overall. A literature review reported that inguinal hernias are 10 times more common in males than in females [9]. 60% of IHs are right-sided, 30% are left-sided and 10–20% are bilateral. The reason why right-sided hernias are found more frequently is that the transition of the right testicle from the abdomen to the scrotum takes longer compared to the left and therefore the closure of the PV on the right side is delayed [10]. In our study, consistent with the literature, the male-to-female ratio was 8.3/1 (89.2% male, 10.8% female). Although IH was more frequently observed on the right side, its prevalence was found to be lower on the right and higher on the left compared to the literature. This finding was not statistically significant (*p* = 0.418).

There are many studies in the literature to explain the mechanisms of IIH formation. For this purpose, environmental and genetic factors have been emphasized frequently. It is generally thought that a multifactorial mechanism is involved [11]. In addition, the molecular mechanism of etiology is still unclear [12]. In the study by Mikami et al. it was reported that IH was rarely observed in some pig species in the pig breeding industry and hereditary factors may be effective on hernia development [13].

It has been found that IH is more common in some families and it has been reported that the disease may have a genetic origin, but there is not enough significant data. Burchart et al. reported that if a mother, father or sibling had a history of IH surgery, the average risk of having IH was 2.4%. They showed that the highest probability was between mothers and daughters (6.01%) [2]. In another study by Burchart et al. positive family history was shown to be an important risk factor for the development and recurrence of IH [14]. In our study, the rate of IH in family history was found to be 8.6% in the study group and 15.4% in the control group, and no statistically significant difference was observed between the groups *(p =* 0.484). Although no gene that may be associated with IH has been reported in the literature, it is also observed that IH has a prominent familial feature.

It is known that connective tissue consisting of collagen, elastic fibers and extracellular matrix (ECM) is important in the pathophysiology of IH. It has been reported that mutations in the *T-box transcription factor 1* (*TBX1*) gene may be responsible for connective tissue diseases and development of congenital diaphragmatic hernia [15]. In a study by Zhang et al. it was reported that DNA sequence variants (DVS) within the *TBX1* gene promoter may change the *TBX1* level and cause the development of IIH as a rare risk factor [16]. In addition, there are studies showing that DVSs within *TBX2* and *TBX3* gene promoters, which are important in embryonic development and found to be expressed especially in connective tissue in humans, may also cause the development of IIH [4, 17].

*E74-like factor 5* (*ELF5*, Entrez Gene 2001, Ensembl ENSG00000135374, UniProtKB Q9UKW6) is an epithelial-specific member of the ETS transcription factor family [18]. *ELF5* is expressed at various embryonic and neonatal sites during embryogenesis, including epithelial exocrine glands, keratinocytes, kidney, prostate, lung and breast [19, 20]. It has been reported that its misexpression may be effective in the etiology of IH by disrupting the specification and differentiation of epithelial cells [21]. In our study, mRNA expression of *ELF5* genes in tissue samples obtained from the patient and control groups was found to be statistically significantly higher in the study group compared to the control group (*p* < 0.001).

*KIF18A* is one of the 45 kinesin proteins found in mice. *KIF18A* has been shown to regulate kinetochore-microtubule binding dynamics by affecting chromosome positioning during mitosis [22]. *KIF18A* has also been reported to attenuate centromere movement through its effect on microtubule pausing [23]. In a study by Luboshits et al. it was found that when treated with the anti-estrogen fulvestrant, *KIF18A*, as a motor protein, can bind to ERα, elicit the non-genomic response of estrogen and activate the extracellular signal regulated kinase (ERK) pathway, and control chromosome compaction and movement during mitosis [24]. Down-regulation of *NPTX1*, a member of the pentraxin family, was observed on efferent channels after treatment with the anti-estrogen fulvestrant in association with altered expression of genes related to ECM organization such as matrix metalloproteinase 7 (MMP7) [25]. These data suggest that estrogen may be associated with normal function of both *KIF18A* and *NPTX1*. Sex hormone de-regulation has been recognized as one of the causes of hernia development [26–28]. Since genetic polymorphisms in both *KIF18A* and *NPTX1* are likely to be linked to estrogen, it was thought that they may also affect estrogen function. Therefore, it has been reported that they may play a role in the development of IH, although the exact molecular mechanism is still unclear [7]. In our study, mRNA expression of *KIF18A* and *NPTX1* genes in tissue samples obtained from the study and control groups revealed that the *KIF18A* value of the study group was statistically significantly higher than that of the control group (*p <* 0.004). There was no statistically significant difference between the groups in terms of *NPTX1* value (*p =* 0.712). Since there was no female patient in the control group, no comment could be made about the effect of estrogen.

Extracellular matrix, which has many physiological and pathological roles, is a highly organized structure [29]. The type XXIII a1 chain encoded by human collagen *COL23A1* is a member of transmembrane collagens, a subfamily of non-fibrillar collagens [30]. A study investigating examining the connective tissue composition in the inguinal region reported lower levels of type 1 collagen and elevated levels of type 3 collagen in cases of IH [31]. In a study by Klinge et al. it was observed that the ratio of type I collagen in the fascia transversalis of patients with IH was significantly decreased compared to the ratio of type III collagen. The tensile strength of tissues depends on the ratio of type I and type III collagen. Accordingly, it was thought that the partial increase in type III collagen (thin, immature fibers) may play a role in the decrease in mechanical strength [32]. Although the sample was taken from the transversalis fascia, 9 out of the 16 cases in the study were pediatric indirect IH. Apoptosis of smooth muscle cells leads to the closure of the PV, with the differentiation of smooth muscle cells into myofibroblasts being a critical step in this process [33]. Myofibroblasts are characterized as contractile cells that secrete collagen [34]. The quantity of smooth muscle within the PV has been shown to influence clinical outcomes such as IH, hydrocele, or undescended testes [35]. In a study by Bellon et al. aberrant collagen metabolism was accepted as one of the main reasons for hernia development and *COL23A1* was found to be associated with hernia development [36]. In our study, mRNA expression of *COL23A1* genes in tissue samples obtained from the study and control groups was found to be statistically significantly higher in the study group compared to the control group (*p <* 0.001).

## Conclusions

This study may be the first study in the literature in terms of genetic factors IH in human tissue samples that we could access. The results of *ELF5*, *KIF18A* and *COL23A1* genes were statistically significantly higher in the study group than in the control group. *NPTX1* values were not significant between the groups. With these results, it can be said that *ELF5*, *KIF18A* and *COL23A1* genes may be counted among the effective factors in PV closure. This study is meaningful in terms of shedding light on larger prospective genetic studies.

## Data Availability

The datasets generated during and/or analyzed during the current study are available from the corresponding author on reasonable request.
